# The War Work of the Voluntary Hospitals

**Published:** 1915-05-01

**Authors:** 


### THE WAR WORK OF THE VOLUNTARY HOSPITALS.

The day of annual meetings and reports for voluntary hospitals is again with us. We have never failed to urge that the annual meeting should be made the occasion for securing the largest possible attendance, and for presenting the work of each hospital in the most telling form, with the aid of the best and really knowledgeable speakers available. The Great War has imposed an immense and increasing volume of work and expenditure upon the greater voluntary hospitals in many parts of the United Kingdom as well as in London. Here, then, is new material which, properly handled, cannot fail to interest all classes, to attract widespread attention, and to secure increased contributions and new supporters. The country is unfortunately not yet alive to the immense services rendered by the voluntary hospitals to the sick and wounded. It is time that everybody should realise that the medical and surgical treatment at, and the staffing of, the voluntary hospitals and kindred institutions which have been and are being organised for the treatment of the sick and wounded all over the country are dependent upon the principal voluntary hospitals, the managers of which are providing members of the medical and surgical staff; they are supplying the nurses and specially training probationers for the work; they are giving up a proportion of the resident medical staff, as well as of the visiting staff; and they are making arrangements for keeping up a continuous supply of the members of the medical profession and of the nursing staff in the establishments where the sick and wounded are to be received, so as to make them efficient. All these services entail increased expenditure, and, important as the Red Cross work necessarily is, that done at and through the voluntary hospitals equals, if it does not even surpass, it in the fact that these great institutions have to treat so many of the severer and more costly cases.

We have said that it is not only the London voluntary hospitals but a large and increasing number of those throughout the United Kingdom which are rendering this yeoman service to the sick and wounded, directly and indirectly. One noticeable proof of this is to be found in the report of the annual meeting of governors of the Norfolk and Norwich Hospital, of which Mr. Charles Larking is chairman. In presenting the report Mr. Larking claimed that the year 1914 must for ever loom large in the history of the world, and had impressed its individuality upon the records of the Norfolk and Norwich Hospital by providing problems of exceptional interest for solution by the governing body. This high claim can be made with equal justice by very many voluntary hospitals, and we hope it will be driven home at the annual meetings everywhere throughout the United Kingdom. If the managers of each voluntary hospital are alert to the claims of the present grave national emergency, they can qualify to take their place in the front rank of institutional workers.

We have not space to do full justice to the Norfolk and Norwich Hospital, but its claims may be tellingly set forth in the briefest words. The managers, by special arrangements and the exhibition of commendable courage, placed 250 beds at the disposal of the military authorities for the reception of the sick and wounded. This action met with some criticism locally on the ground that the hospital must cease in very large measure to do its normal work. This point was disposed of by Dr. Barton, the senior physician, who stated that the exact contrary was the case, for the waiting list for ordinary in-patients was smaller now than it had been for years. During the quarter ending March 31, 1915, the average daily number of ordinary in-patients was 257, whilst the daily average number of beds occupied—that is, of ordinary in-patients—in the year before the war was only 180. The war is stimulating people to greater intelligence in many directions. We hope it may make the local and other critics of our voluntary hospitals remain silent at least until they have facts upon which to rest their criticism. The Hospital of January 13, 1914, gave a full report of this institution, in the course of which stress was laid upon the fact, which the 1915 annual report emphasises, that the county and city of Norwich have the immense advantage of possessing officials, who are animated by the spirit of personal service for the sick to a very unusual extent, and to this is no doubt due the splendid efficiency of its administration.

